# T2*-weighted MRI technique for visualization of RF ablation lesions

**DOI:** 10.1186/1532-429X-18-S1-O128

**Published:** 2016-01-27

**Authors:** Eugene Kholmovski, Ravi Ranjan, Nathan Angel, Nassir F Marrouche

**Affiliations:** 1 UCAIR, Department of Radiology, University of Utah, Salt Lake City, UT USA; 2 CARMA Center, University of Utah, Salt Lake City, UT USA

## Background

LGE-MRI is widely used to assess cardiac RF ablation lesions. However, LGE-MRI requires contrast injection and the appearance, dimensions and visibility of lesions in LGE-MRI noticeably change with time after ablation and time after contrast injection. Recently proposed non-contrast T1-weighted (T1w) technique is only applicable to visualize acute (< 3 days) RF lesions. The main goal of this study was to develop and validate a non-contrast MRI technique for assessment of sub-acute (> 3 days) RF ablations.

## Methods

Non-contrast T2*-weighted (T2*w) MRI technique for RF lesion visualization has been implemented. This technique exploits the difference in T2* relaxation between normal and ablated myocardium. Reduction in T2* relaxation time of ablated myocardial tissues is caused by the transformation of hemoglobin into hemosiderin from ruptured and obstructed blood vessels as a result of RF ablation. To validate this technique, RF ablations were performed in 6 canines using ThermoCool catheter (Biosense Webster) at 30 Watts for 30 seconds. Imaging studies were performed on a 3T scanner (Verio, Siemens HealthCare) at 0, 1, 4, and 8 weeks post-ablation. Study protocol included T1w, T2*w, and LGE scans with a resolution of 1.25 × 1.25 × 2.5 mm and T1, T2 and T2* mapping.

## Results

**Figure 1:**
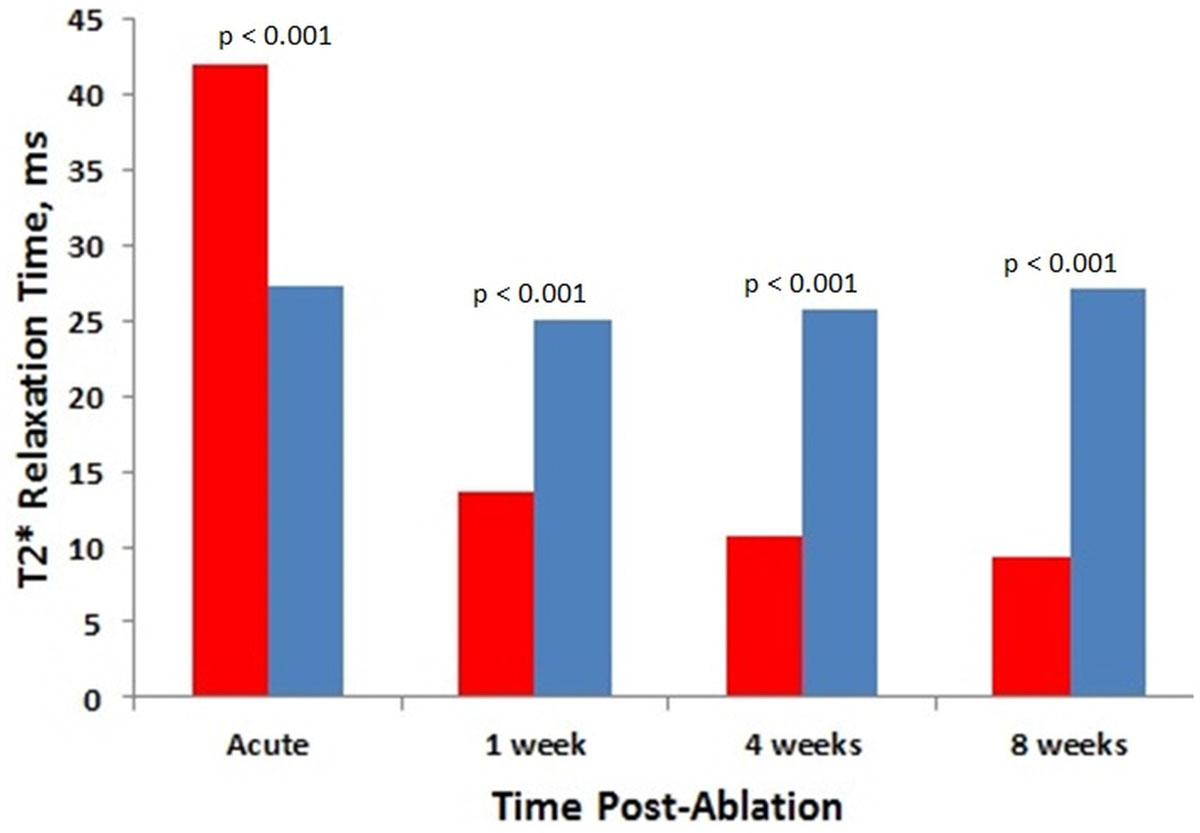
T2* relaxation time of ablated and normal myocardium vs. time after ablation. Blue bars - normal myocardium, red bars - RF ablation lesions.

Dependence of T2* relaxation time of ablated and normal myocardium on time after ablation is shown in Fig. 1. T2* of normal myocardium was similar for all post-ablation studies (p = N.S.). For acute (0 week) studies, T2* relaxation time of ablated regions (42.0 ± 8.8 ms) was significantly higher (p < 0.001) than T2* for normal myocardium (27.4 ± 3.7 ms). This observation may be explained by the presence of severe edema at the ablated regions. T2* time of RF ablations significantly reduced with time after ablation (p < 0.05) and it was significantly lower than T2* of normal myocardium at 1, 4, and 8 weeks after ablation (p < 0.001).

**Figure 2:**
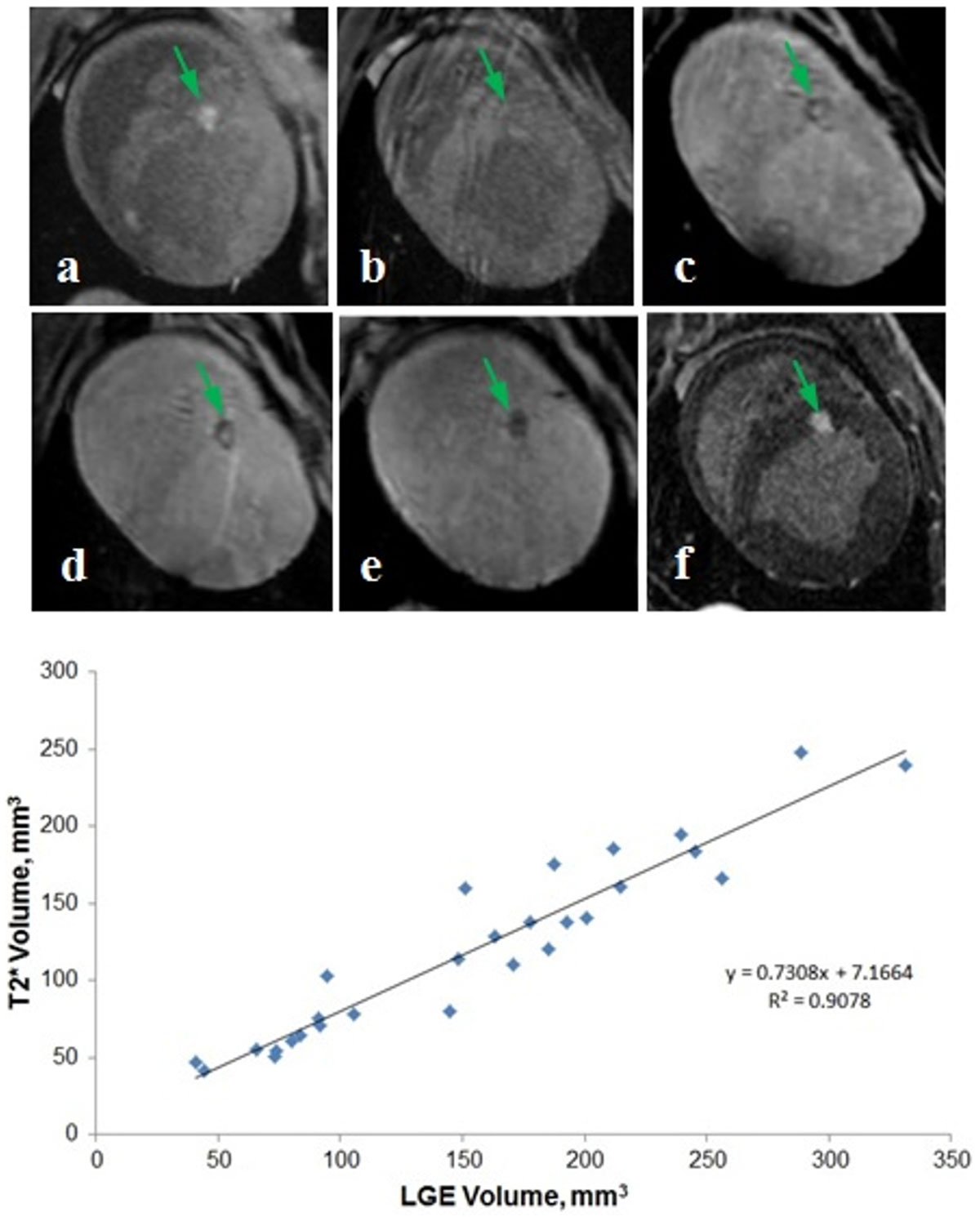
**Top Panel - Representative images of RF ablation lesion**. (**a-b**) non-contrast T1w acquired (**a**) acutely and (**b**) 1 week post-ablation; (**c-e**) non-contrast T2*w acquired (**c**) 1, (**d**) 4, and (**e**) 8 weeks post-ablation; (**f**) LGE acquired 8 weeks post-ablation. **Bottom Panel** - Volume of RF ablation lesions from LGE-MRI vs. volume of the lesions from T2*w. LGE and T2*w were acquired 8 weeks post-ablation.

Representative T1w, T2*w, and LGE images are shown in Fig. 2, top panel. All RF lesions (n = 28) were detectable on non-contrast T1w images acquired acutely. Lesion visibility in non-contrast T1w MRI was considerably reduced 1 week post-ablation. Visibility of lesions in T2*w images improves with time after ablation. Lesions have hypointense boundaries in T2*w images acquired 1 and 4 weeks post-ablation. Whole lesions are hypointense in T2*w images acquired 8 weeks post-ablation.

Strong correlation (R^2^ = 0.908) between lesions volumes estimated from LGE and T2*w images acquired 8 weeks post-ablation was found (Fig. 2, bottom panel). Lesion volume from T2*w scans was about 27% smaller than lesion volume from LGE scans.

## Conclusions

T2* relaxation time of cardiac RF ablation lesions significantly reduces with time after ablation. Non-contrast T2*w technique can be used to visualize sub-acute RF ablations as early as a week post-ablation. Visibility of the lesions in T2*w image considerably improves with time after ablation as T2* relaxation time of the lesions becomes shorter.

